# Nanoscale graphene Hall sensors for high-resolution ambient magnetic imaging

**DOI:** 10.1038/s41598-019-50823-8

**Published:** 2019-10-08

**Authors:** David Collomb, Penglei Li, Simon J. Bending

**Affiliations:** University of Bath, Claverton Down, Bath, BA2 7AY United Kingdom

**Keywords:** Two-dimensional materials, Nanosensors

## Abstract

A major challenge to routine non-invasive, nanoscale magnetic imaging is the development of Hall sensors that are stable under ambient conditions and retain low minimum detectable fields down to nanoscale dimensions. To address these issues we have fabricated and characterised chemical vapour deposition (CVD) graphene Hall sensors with wire widths between 50 nm and 1500 nm, in order to exploit the high carrier mobility and tuneability of this material. The measured Hall voltage noise is in good agreement with theoretical models and we demonstrate that minimum detectable fields at fixed drive current are lowest in the vicinity of the charge neutrality point. Our best performing deep sub-micron sensors, based on a wire width of 85 nm, display the excellent room temperature resolution of 59 µT/√Hz at a dc drive current of 12 µA and measurement frequency of 531 Hz. We observe a weak increase in minimum detectable field as the active sensor area is reduced while the Hall offset field is largely independent of size. These figures-of-merit significantly surpass prior results on larger probes in competing materials systems, with considerable scope for further optimisation. Our results clearly demonstrate the feasibility of using CVD graphene to realise very high spatial resolution nanosensors for quantitative room temperature magnetic imaging.

## Introduction

Magnetic field sensors are used for a very wide variety of purposes, including but not limited to; biosensing, instrumentation and process calibration as well as high precision magnetic field mapping such as Scanning Hall Probe Microscopy (SHPM) and magnetic susceptometry^1–5^. Although there are many different approaches to magnetic sensing, Hall-effect sensors have frequently been employed due to their high magnetic field sensitivities, quantitative linear response, non-invasive performance and fabrication versatility. This allows them to be used in a range of applications where other semi-quantitative and potentially invasive sensor types, such as magnetic force microscopy (MFM) cantilevers, may not meet requirements^6^. They are also much more compact and simple to use than the recently developed diamond Nitrogen vacancy (NV) centre magnetic microscope, which requires precisely fabricating a single crystal diamond with an NV center at the apex of an AFM tip as well as additional lasers and microwave excitation^7^. The fabrication of Hall probes based on nanoscale wire widths allows high spatial resolution mapping applications to be realised. This requirement is increasingly in demand due to the rapid miniaturisation of modern technologies, for example ultra-high density magnetic storage media or magnetic domain-wall racetrack memory. However, such probes have a much broader range of potential imaging applications including combined high-resolution topographic and magnetic imaging of vortices in superconductors and domains/domain walls in ferromagnetic films based on SHPM with scanning tunnelling microscopy (STM) or atomic force microscopy (AFM) surface tracking. Through the addition of field excitation coils they can also be used to perform highly local magnetic susceptometry.

To attain higher spatial resolution for these smaller scale applications, the active area of the Hall probe must be reduced while retaining sufficiently low minimum detectable fields. In Table 1 we summarise the performance of previously reported Hall probes, including the estimated spatial resolution the sensor would have in magnetic imaging applications.

**Table 1:** A comparison of previous Hall probe architectures at room temperature and low measurement frequencies.

Hall material	*R*_*H*_ (Ω/T)	*I*_*H*_ (µA)	*f* (Hz)	*B*_*min*_ (µT/√Hz)	*X*_*min*_ (µm)
GaAs quantum well^8^	1100	100	277	1000	0.8
InSb thin film^1^	370	100	200	0.72	0.5
InSb thin film^9^	300	300	N/A	0.08	1.25
Bi thin film^10^	1.81	73	30	900	0.1
Bi thin film^11^	4	40	1000	80	0.05
Epitaxial graphene^36^	640	10	3300	49.3	0.5
CVD graphene^18^	800	100	3000	0.5	15
CVD graphene^19^	2093	200	3000	0.1	50
CVD graphene (this work)	140	12	531	59	0.085

The figures of merit include, when applicable, the Hall coefficient, *R*_*H*_, the drive current, *I*_*H*_, the measurement frequency, *f*, the minimum detectable field, *B*_*mim*_, and the estimated spatial resolution based on the wire width, *X*_*min*_.

Probes based on GaAs heterostructures are the material of choice for low temperature imaging but, as reflected in Table 1, their electronic properties deteriorate undesirably at room temperature^8^. In addition, edge depletion effects make it very challenging to achieve suitably high spatial resolution devices on the order of hundreds of nanometres or below, with no Hall crosses yet demonstrated below 100 nm^8^. High quality epitaxial growth of InSb-based probes is challenging and active layers are typically buried ≥50 nm below the epilayer surface^1^. The device reported in ref.^1^ was also fabricated in a 320 nm thick InSb film leaving little scope for further reduction in size^1,9^. This is detrimental for applications such as SHPM, where the active layer must be as close to the sample surface as possible to achieve the highest spatial resolution. Bismuth probes have demonstrated reasonable 300 K resolutions, yet these suffer from poor chemical and mechanical stability and the reproducible growth of Bi films is challenging^10,11^, making them unsuitable for extended operation under ambient conditions.

In contrast, graphene’s low carrier density, tolerance to nanoscale pattering, mechanical and chemical stability and its unique band structure, whereby massless Dirac Fermions exhibit extremely high room temperature mobilities, make it an ideal contender for high-resolution nanoscale Hall probes^12^. Being atomically thin it also allows the active probe to get extremely close to samples under study, enabling very high spatial resolution mapping. Looking towards the more routine fabrication of graphene-based Hall devices, chemical vapour deposition (CVD) holds promise as a facile growth technique for the scalable fabrication of large arrays of sensors^13,14^. In addition, once transferred to an appropriate insulating substrate, graphene is a much easier material to pattern at the nanoscale than alternative Hall probe materials; a lithography mask can readily be used to transfer a Hall cross pattern by etching in a simple O_2_ plasma. Monolayer CVD graphene is now readily available and is the obvious choice for the scaleable production of graphene-based Hall effect sensors^15^. Combined with a back gate dielectric such as SiO_2_, the carrier density and type can also be tuned, allowing an additional ‘tool’ for optimising minimum detectable fields that has not previously been available.

Micrometer-sized graphene Hall probes have been extensively studied in recent years, showing impressive figures-of-merit, with some of the best reported probes shown in Table 1. However, investigations of Hall sensors with nanoscale active areas, i.e., <1 µm wire widths as required for high spatial resolution magnetic imaging, have not been reported^16–19^. Studies of the mobility of graphene devices with nanoscale dimensions have shown a decrease from ~3000 cm^2^/Vs for probes greater than 100 nm to <200 cm^2^/Vs for probes smaller than 20 nm. A rapid decrease in mobility due to edge scattering tends to set in below 60 nm^20^.

However, we demonstrate that the figures-of-merit of CVD graphene nanosensors significantly surpass those based on competing materials with much larger dimensions. We have studied the influence of wire width, *w*, on the minimum detectable field, *B*_*min*_, and systematically investigated the impact of increasing drive currents and varying carrier density and carrier type. These critical variables have previously either been overlooked or studies have produced inconclusive results^16–19^. We establish the practical limitations for nanoscale CVD Hall sensors and ascertain the optimal measurement conditions for real-world high spatial resolution imaging applications.

### Estimation of the minimum detectable field

**Figure 1:**
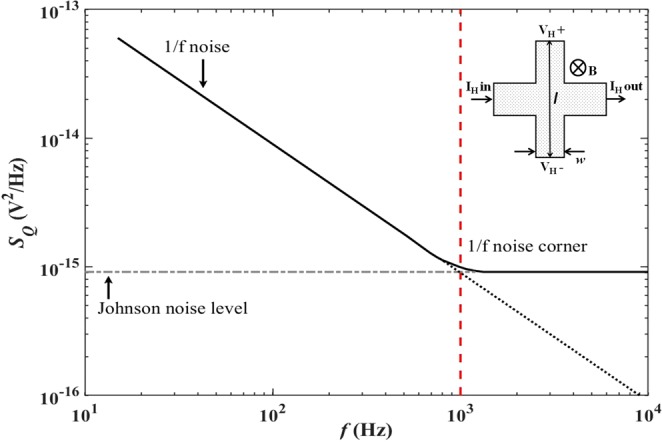
Schematic frequency dependence of the noise power in Hall devices with extrapolated lines indicating the behaviour of Johnson noise and 1/f noise below and above the 1/f noise corner. The inset shows a sketch of a typical Hall cross indicating the current leads, Hall voltage leads and their dimensions.

The Hall voltage from our sensors contains several intrinsic sources of noise with different characteristic frequency dependencies. At low frequencies this is dominated by “1/f noise” whose power density is inversely proportional to the measurement frequency. The origin of 1/f noise in graphene Hall devices has been the subject of several experimental studies^21–23^, and it is widely accepted that it arises from ‘exchange noise’ due, for example, to carrier capture and release at traps in the SiO_2_ gate dielectric leading to fluctuations in the carrier density, *n*^24^. A second contribution arises from ‘configuration noise’ due to rearrangement of adjacent trapped charges which modify the disorder potential landscape, leading to fluctuations in the carrier mobility, *µ*^24,25^. However, as the measurement frequency is increased the 1/f noise power drops below the frequency-independent Johnson noise power at a characteristic noise corner as plotted schematically in Fig. 1. The latter arises due to the thermally excited motion of charge carriers within the sensor, and its magnitude depends on the resistance of the Hall voltage leads.

The low frequency (below the 1/f noise corner) transverse voltage noise power limit for our 2D Hall sensors can be described using the conductivity fluctuation model proposed by L. K. J. Vandamme *et al*.^26^

1 $${S}_{Q}^{1/f}=\,\frac{{{I}_{H}}^{2}{\rho }_{xx}^{2}\alpha }{AFfn},$$

where *I*_*H*_ is the drive current, *ρ*_*xx*_ the longitudinal resistivity, *α* a dimensionless constant (expected to be on the order of 10^−3^), *A* is the contact-free surface area, *F* is a geometric factor (~1 for a cross with the aspect ratio of 5:1 used here) and *f* is the measurement frequency.

Well above the 1/f noise corner the Johnson noise power per unit bandwidth is given by

2 $${S}_{Q}^{J}=4{R}_{vv}{k}_{B}T,$$

where *R*_*vv*_ is the resistance between the Hall voltage contacts and T is the temperature. Comparing Eqs (1) and (2) we see that 1/f noise depends strongly on the Hall probe current, I_H_, while the Johnson noise is independent of it. Hence the position of the 1/f corner (and the dominant noise mechanism) varies with the sample current, moving to higher frequencies as the current is increased. As a consequence the minimum detectable field at a fixed measurement frequency will depend strongly on the current density and can be defined by

3 $${B}_{min}=\frac{\sqrt{{S}_{Q}\Delta f}}{{R}_{H}{I}_{H}},$$

where Δf is the measurement bandwidth and *R*_*H*_ is the Hall coefficient given by

4 $${R}_{H}=\frac{1}{nq}=\frac{1}{{I}_{H}}\frac{\delta {V}_{H}}{\delta B}.$$

Here *V*_*H*_ is the measured Hall voltage and *B* is the applied magnetic field. Using the standard expression for the resistivity, ${\rho }_{xx}=1/ne$*μ*, in the low frequency limit described by Eq. (1) this yields

5 $${B}_{min}^{1/f}=\frac{1}{\sqrt{n}e{\mu }}\sqrt{\frac{\alpha \Delta f}{AFf}},$$

while in the high frequency limit defined by Eq. (2) we find

6 $${B}_{min}^{J}=\frac{1}{{I}_{H}}\sqrt{\frac{n}{\mu }}\sqrt{\frac{4le{k}_{B}T\Delta f}{w}},$$

where *l* is the length and *w* the width of the Hall voltage contacts as illustrated in the inset of Fig. 1.

In practice it is well established that the carrier mobility in CVD graphene implicitly depends on the carrier density, *n*^27^. Assuming *µ*~ 1/*n*^*η*^ we find the following limiting dependencies on carrier density, current and frequency in both limits.

7 $${B}_{min}^{1/f}\propto \frac{{n}^{(\eta -0.5)}}{\sqrt{f}},$$

and

8 $${B}_{min}^{J}\propto \frac{{n}^{(\eta /2+0.5)}}{{I}_{H}}.$$

In practice, upon fitting transconductance curves for all our devices we find *η* ~ 0.6, suggesting that scattering by neutral impurities is dominant in our structures^28^. Hence we expect the coefficient of carrier density to lie somewhere between the limiting values of ~0.1 and ~0.8, while the coefficient of Hall current will lie between 0 and −1.

## Results and Discussion

### Hall sensor transfer characteristics

**Figure 2:**
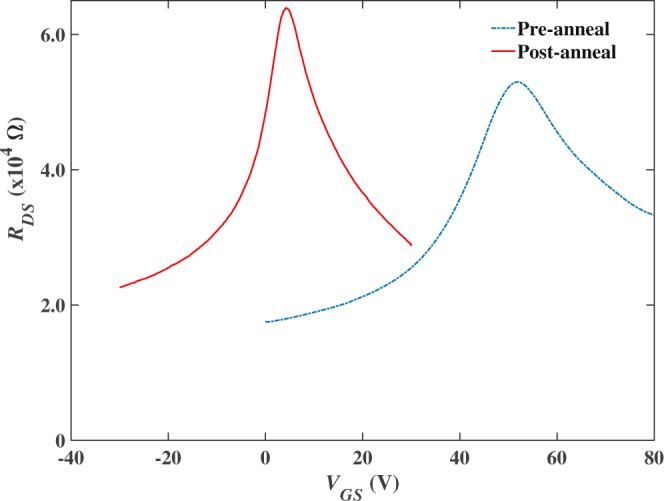
Resistance (*R*_*DS*_) versus back gate voltage (*V*_*GS*_) for a 1000 nm cross before and after the post-fabrication anneal, showing a pronounced downwards shift of the CNP.

Transfer characteristics (R_DS_(V_GS_)) of the graphene Hall crosses were measured in 2-lead configuration across pairs of Hall voltage leads. A typical curve is shown in Fig. 2 for a CVD graphene Hall cross based on a 1000 nm wire width immediately after fabrication. All of our devices were found to be quite heavily hole doped (n_h_ ~ 1.7 × 10^16^–5.0 × 10^16^ m^−2^), with the charge neutrality point (CNP) up to and sometimes beyond V_GS_ = +100 V before post-fabrication annealing.

We estimate a field-effect mobility for our Hall cross devices from ${\mu }_{FE}=\frac{t}{{{\epsilon }}_{r}{{\epsilon }}_{0}}\frac{d{\sigma }_{DS}}{d{V}_{GS}}$, where *σ*_*DS*_ is the conductivity determined from transfer curves for the known geometry of the graphene segment, *t* is the gate oxide thickness and *ε*_*r*_ the relative permittivity of the SiO_2_ gate oxide. The extracted mobilities range from 5100 cm^2^/Vs for a 1000 nm wire width cross down to 840 cm^2^/Vs for a 50 nm cross measured after annealing in the hole-doped regime at *V*_*g*_ − *V*_*DP*_ = +7 V. The lower mobility of nanoscale devices is a consequence of the increasing role of edge disorder as the perimeter: surface area ratio increases, and is also seen in graphene nanoribbons^20^. An asymmetry between hole-doped and electron-doped regimes was observed in many devices and ascribed to the different scattering mechanisms affecting the charge carriers in graphene, such as the presence of charged-defects from transfer and fabrication processes which lead to the preferential scattering of one carrier type^29^. Another potential source of the carrier-type asymmetry arises from the metal contacts; charge transfer from the metal to graphene can lead to p-p or p-n junctions^30^.

### Hall coefficient and Hall voltage noise characterisation

**Figure 3:**
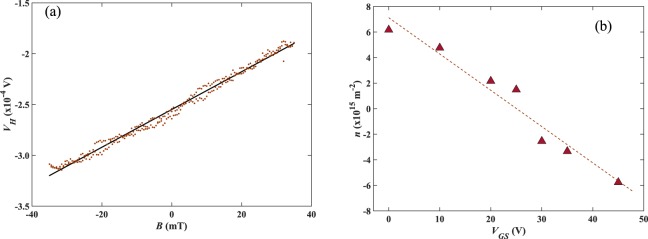
(**a**) A typical Hall voltage versus magnetic field trace at I_H_ = 10 µA for a 700 nm cross. (**b**) The carrier density, *n*, calculated from measurements of *R*_*H*_ as a function of *V*_*GS*_ for a 700 nm Hall cross with the CNP located at *V*_*D*_ = 27 V. The dotted line represents a linear fit to Eq. (9).

It has been shown in previous studies that the maximum magnetic sensitivity is found just either side of the CNP^31^. The carrier density is calculated from Eq. (4) using the Hall coefficient obtained from traces of the Hall voltage measured as a function of magnetic field, such as in Fig. 3(a).

Treating graphene and the doped Si substrate as the plates of a capacitor the surface charge density induced by a back gate voltage can be described by the parallel plate capacitor equation. For the general case of graphene with extrinsic doping this yields

9 $$n=\frac{{{\epsilon }}_{r}{{\epsilon }}_{0}}{te}({V}_{GS}-{V}_{D}),$$

where *t* is the thickness of the gate oxide with relative permittivity *ϵ*_*r*_ and *V*_*D*_ is the gate voltage at the CNP. The effect of the graphene quantum capacitance can be ignored at room temperature since it is several orders of magnitude larger than the classical gate oxide capacitance in series with it. Figure 3(b) confirms that the net carrier concentration depends linearly on the back gate voltage, as expected.

**Figure 4:**
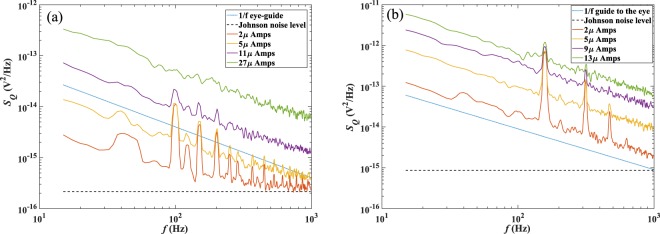
Hall voltage noise power, *S*_*Q*_, in the range 1 Hz to 1 kHz at various dc drive currents for a 1500 nm (**a**) Hall cross and for a 85 nm (**b**) Hall cross with no applied back gate voltage. The dashed lines indicate the calculated Johnson noise level for the devices. Both structures were measured at similar carrier densities of n_h_ ~ 2.5 × 10^16^m^−2^. Peaks in the very low current spectra arise from 50 Hz (and higher harmonic) pick-up from the 230 V ac mains supply, but since these occur at discrete frequencies they do not influence the underlying 1/f noise dependence.

Figure 4(a) shows Hall voltage noise power as a function of frequency in the range 1 Hz to 1 kHz for a 1500 nm Hall cross at several different fixed drive currents. For all currents the spectrum is dominated by 1/f noise at low frequencies, with a 1/f noise corner of about 300 Hz at the lowest 2 µA drive current. The 1/f noise level increases substantially as the drive current is increased and the corner frequency rapidly moves above our maximum measurement frequency. For comparison Fig. 4(b) plots the noise power per unit bandwidth for a much smaller 85 nm Hall sensor at several drive currents, when in all cases we observe a 1/f noise spectrum across our entire measurement window. Comparing the noise spectra of smaller and larger devices, we note that larger probes generally have lower corner frequencies than smaller ones as expected.

Despite being the subject of several experimental studies, there does not appear to be a consensus on how the low-frequency noise depends on carrier density in graphene, and several different relationships have been reported^21–23^. For devices which are fully in the 1/*f* limit, using known *ρ*_*xx*_ values, we calculate the value of *α* in Eq. (1) to be on the order of ~10^−3^ as expected.

**Figure 5:**
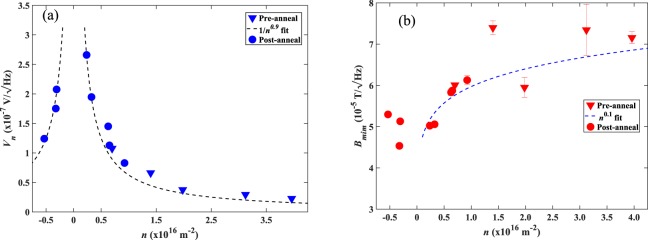
(**a**) The measured Hall voltage noise at 531 Hz with a 2 µA drive current for a 400 nm wire width cross at various carrier concentrations in both the electron- and hole-doped regimes. (**b**) The minimum detectable field for the same cross estimated by inputting the measured Hall-coefficient (from Hall voltage traces such as that in Fig. 3(a)) and the measured Hall voltage noise into Eq. (3). The results are compared with the expected *n*^0.1^ dependence in the “1/f noise-dominated limit” (dashed line, see text) for the data in the hole-doped regime.

Figure 5(a) shows that the measured Hall voltage noise of our devices as a function of carrier concentration tends to diverge as the CNP is approached from above or below, and both pre- and post-annealed datasets appear to sit on a single universal curve. Figure 5(b) is a plot of the carrier density dependence of *B*_*min*_ for the same 400 nm Hall cross with a fixed current of 2 µA, showing that away from the CNP the data are well described by the *n*^0.1^ dependence predicted by Eq. (7) in the 1/f noise-dominated limit. Empirically we find that the lowest minimum detectable fields at a constant drive current are located at about ±2.5 × 10^15^ m^−2^ either side of the CNP.

### Optimisation of *B*_*min*_ via the drive current

**Figure 6:**
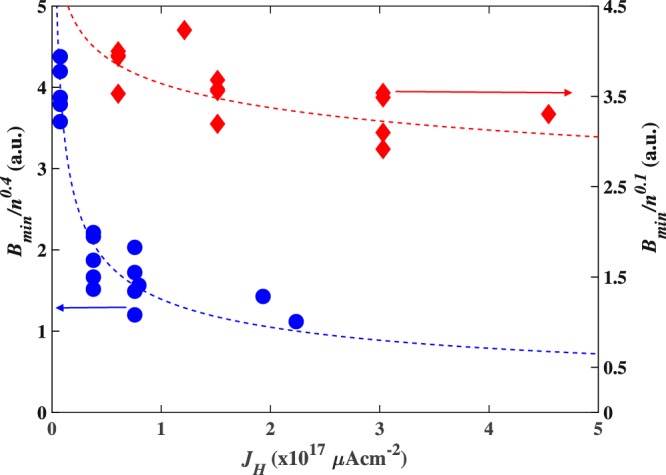
Estimated minimum detectable fields scaled by *n*^0.4^ for an 800 nm (left axis) and *n*^0.1^ for a 100 nm (right axis) wire width probe at 531 Hz as a function of drive current density. The dotted lines show a *1/J*_*H*_^0.41^ fit for the 800 nm probe and a *1/J*_*H*_^0.11^ fit for the 100 nm probe.

In previous Hall probe architectures, it has been shown that *B*_*min*_ could be optimised by increasing the drive current (or current density, *J*_*H*_). For large sensors, that are initially closer to the thermal noise limit, *B*_*min*_ starts to increase again if the current is raised beyond this optimum point^1,6^. Figure 6 plots *B*_*min*_ at 531 Hz as a function of *J*_*H*_ for two different sized probes after scaling by a fractional power of *n* in order to collapse the data onto a single universal curve. For the 100 nm probe the same n^0.1^ density noise scaling is found as for the 400 nm probe in Fig. 5. However the larger 800 nm probe is much closer to being in the Johnson noise limit and, as expected, we find a stronger n^0.4^ scaling in this case. Both data sets show a decrease in *B*_*min*_ as *J*_*H*_ increases from zero, with a noticeably weaker dependence in the smallest sensor. We have quantified this by making fits to the data of the form 1/J_H_^β^ (dashed lines), and find β = 0.11 for the 100 nm probe and β = 0.41 for the 800 nm probe. In practice the Hall voltage offset saturated our low noise preamplifier at higher current densities before the regime of increasing noise levels could be accessed in the larger probe.

*B*_*min*_ in all our CVD graphene devices appears to be well-described by *1/J*_*H*_^*β*^, with values of *β* ranging from 0.87 for the largest Hall crosses to 0.08 for the smallest sensors studied. This is broadly consistent with our expectations that it will depend as 1/*I*_*H*_ in very large sensors in the thermal noise limit (Eq. (8)) and be independent of *I*_*H*_ in very small sensors dominated by 1/f noise (Eq. (7)). In practice the trapping of carriers in the gate dielectric is a thermally activated process which is strongly influenced by the temperature of the carrier population. At high current densities carriers can be heated well above the lattice temperature leading to much higher trapping rates, increasing the noise power at a given frequency and shifting the 1/*f* noise corner up to higher frequencies^32^. Hence, even very large sensors should be shifted towards the 1/f noise-dominated regime as the current density is increased.

Upon increasing the drive current, we have obtained our lowest minimum detectable field of 6 µT/√Hz at 30 µA and 531 Hz for a 1000 nm wire width cross, significantly surpassing values achieved in prior works on similar-sized devices^33^. Since *B*_*min*_ does not exhibit saturation at the highest current densities used there is evidently still scope for reducing the minimum detectable field still further if the offset voltage could be reduced.

### Wire width-dependence of minimum detectable field

**Figure 7:**
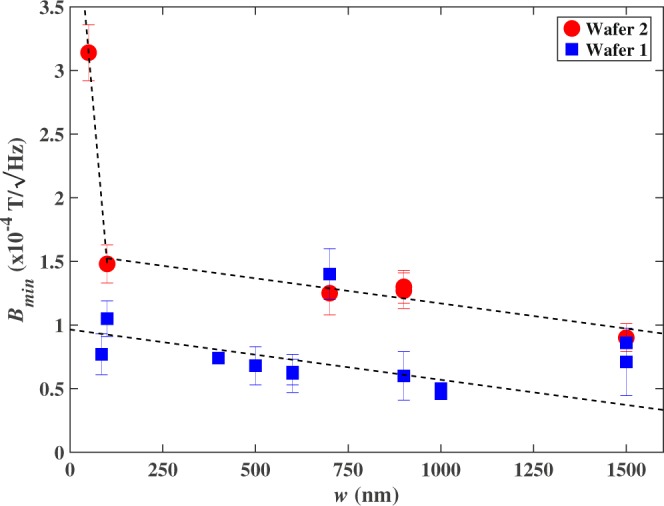
Estimated minimum detectable field as a function of wire width for a fixed drive current of 2 µA at 531 Hz and similar values of *R*_*H*_ in the range 140–180Ω/T. Solid circles and squares indicate samples fabricated from different CVD graphene wafers. Dashed lines are given as a guide to the eye.

Figure 7 indicates that minimum detectable fields for a fixed drive current show a gradual increase as the Hall probe size decreases down to about 85 nm. Results for two different CVD graphene wafers have been compared to indicate the variability that can exist for different CVD growth runs and wet transfers. Otherwise identical devices fabricated from wafer 2 had higher lead-to-lead resistances than wafer 1 for similar carrier densities, leading to higher noise levels and minimum detectable fields. In addition to different concentrations of contaminants that become adsorbed on the graphene during transfer we attribute this to different densities of grain boundaries, other defects and localised impurities formed during CVD growth. The rapid increase in *B*_*min*_ observed in the 50 nm Hall cross may indicate that edge scattering is starting to dominate the noise in such small sensors, and would be consistent with the conclusions of previous studies showing that the mobility of graphene nanoribbons begins to drop more rapidly below ~60 nm wire widths^20^.

Despite the gradual reduction in performance as the Hall cross size is reduced, our best performing deep sub-micron cross with an 85 nm wire width, exhibits a minimum detectable field of 59 µT/√Hz at a drive current of 12 µA at 531 Hz, with significant room remaining for improvement by increasing the drive current. This surpasses the optimum figure-of-merit for prior Bismuth Hall probes (~900 µT/√Hz for a 100 nm probe) by more than an order of magnitude^10^, paving the way for graphene-based Hall probes to become the state-of-the-art in high spatial resolution room temperature magnetic imaging applications.

### Hall offset resistance in graphene devices

An aspect that is frequently overlooked in investigations of Hall sensors is the offset resistance, *R*_*off*_. This is the Hall voltage output when no magnetic field is present, arising due to misalignment of the Hall voltage contacts and inhomogeneous current flow in the active sensor area^34^. In practice it is highly desirable to minimise this to avoid saturating low noise preamplifier stages. In addition, by mixing the longitudinal resistance into the Hall voltage, the offset resistance contributes substantial new sources of noise that ultimately limit the best achievable minimum detectable fields.

**Figure 8:**
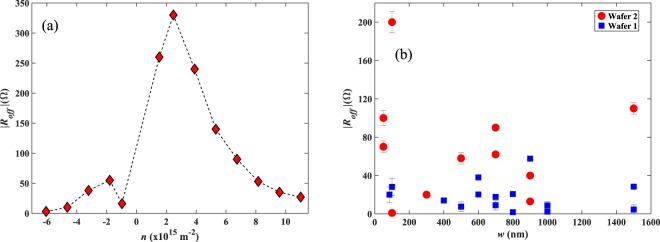
(**a**) Hall offset as a function of *n* for a 700 nm cross. The dashed line is a guide to the eye. (**b**) Hall offset as a function of device size at similar values of *R*_*H*_ in the range 140–180Ω/T and fixed drive currents of 10 µA for two different graphene wafers.

Figure 8(a) shows offset voltages were broadly observed to increase in inverse proportion to the carrier density, while around the CNP we frequently observe an abrupt discontinuous change with a rather different behaviour for electron and hole carrier types. In our CVD graphene sensors the offset resistance appears to predominantly arise from spatially inhomogeneous current flows linked to the presence of grain boundaries, wrinkles introduced during transfer, multilayer regions or other defects. The fact that the offset resistance roughly scales with the Hall coefficient suggests that the degree of inhomogenity is approximately preserved as the carrier concentration is changed with a back gate. The different behaviour for the two carrier types is in part to be expected since potential minima for holes become potential maxima for electrons upon crossing the CNP. Certainly, it indicates that the level of current inhomogeneity for holes can be appreciably different to that for electrons.

The potential invasiveness of our Hall probes can be characterised by estimating the self-field using Ampere’s law. For a 85 nm probe carrying a 12 µA current, the self-field is estimated to be ~0.5 G at a distance of 50 nm, which is comparable to the earth’s magnetic field and hence relatively non-invasive. If the dominant cause of the offset resistance in our devices was linked to grain boundaries, one would expect a substantial reduction in sensors that are very much smaller than a characteristic grain size in CVD graphene (~10 µm). However, this is not supported by the data for *R*_*off*_ as a function of wire width in Fig. 8(b), which shows no strong evidence for a systematic size dependence. This suggests that the current inhomogeneity still occurs on a length scale much finer than the smallest sensors fabricated here, possibly as a result of disorder introduced via graphene transfer to the SiO_2_ substrate.

## Conclusion

We have demonstrated that CVD graphene can be used to fabricate nanoscale Hall sensors for state-of-the-art high spatial resolution magnetic imaging. Their minimum detectable fields can be optimised by tuning the carrier concentration with a back gate to values of about ±2.5 × 10^15^ m^−2^, just either side of the charge neutrality point. Hall probe performance can be still further improved by increasing the drive current, leading to figures-of-merit that significantly surpass those of competing Hall sensor structures, e.g., a minimum detectable field of 59 µT/√Hz for an 85 nm wire width sensor at 531 Hz at a 12 µA drive current. Advances in highly quality CVD graphene growth and transfer methodologies, combined with the rather simple fabrication processes for deep sub-micron probes, makes them extremely attractive for high-resolution magnetic imaging applications such as SHPM and magnetic susceptometry. A dramatic improvement in the performance of exfoliated graphene devices after encapsulation is well documented and we expect to be able to achieve much better figures-of-merit in sensors encapsulated with hBN^19^, although achieving this with CVD graphene remains a major challenge. The introduction of these improvements should make nanoscale graphene Hall sensors the quantitative tools of choice for high resolution magnetic imaging under ambient conditions.

## Experimental Section

### Hall cross array fabrication

**Figure 9:**
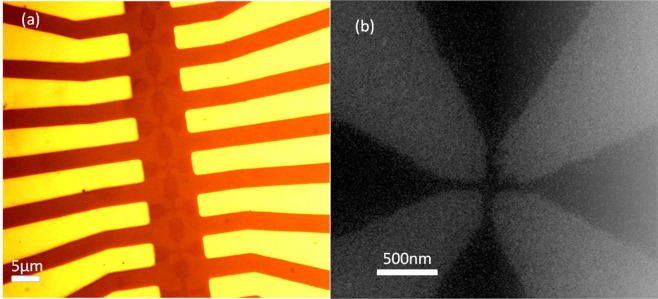
(**a**) Optical micrograph showing part of a fabricated Hall cross array (darker brown) and the inner Cr/Au contact leads (gold). (**b**) An SEM image of an 85 nm CVD graphene Hall cross.

Hall probe arrays were fabricated from CVD graphene purchased from Graphene Supermarket, which had been grown on a copper foil and wet-transferred onto a highly-doped Si substrate with a 285 nm thick SiO_2_ surface gate oxide. An optical micrograph of a CVD graphene Hall cross array and the Cr/Au inner contact leads is shown in Figure. 9(a), while an SEM micrograph of a deep sub-micron Hall cross based on an 85 nm wire width is shown in Figure. 9(b). Hall crosses based on the intersection of wires of widths in the range 50–1500 nm, with length-to-width aspect ratios of five, have been systematically investigated.

Contact pads were defined by direct-write laser lithography in Shipley S1813 optical photoresist. The exposed CVD graphene in the developed window was then ICP-etched in an Oxygen plasma, followed by the deposition of 20 nm of Cr and 250 nm of Au by electron beam evaporation and lift-off. Inner contact leads were then defined by laser lithography, followed by the deposition of 5 nm of Cr, 70 nm of Au and lift-off.

To achieve the best lateral resolution, the fabrication of nanoscale Hall probes made use of a bilayer Poly(methyl methacrylate) (PMMA)/Hydrogen Silsesquioxane (HSQ) electron beam resist as an etch mask. PMMA was first spin coated at a thickness of 100 nm, and baked at 150 °C for 15 minutes. A 2% solution of negative tone HSQ from Dow corning was then spin coated to form a 30 nm thick layer and baked for 5 minutes at 150 °C. A 30 kV field emission Hitachi S-4300 scanning electron microscope (SEM) integrated with the Raith ELPHY Plus electron beam lithography system was used to pattern the Hall cross arrays in HSQ with an exposure dose of 500 µCcm^−2^. After development in Tetramethylammonium hydroxide (TMAH) the unprotected PMMA and graphene were ICP-etched away in an O_2_ plasma. The HSQ mask was subsequently lifted-off by dissolving the PMMA layer in acetone under light sonication.

### Post anneal treatment

We utilise a post-fabrication anneal in inert Argon gas at 200 °C for 6 hours to reduce extrinsic doping due to any low molecular weight contaminants adsorbed on the graphene during the fabrication process. Annealing temperatures up to 400 °C have been widely report in the literature^35^, but we find that such high temperatures lead to hardening of the gold contact pads making them very difficult to wire bond. This post-fabrication annealing step successfully reduces the extrinsic (hole) doping level, shifting the graphene CNP by up to −60V.

### Device characterisation

A portion of the oxide layer on the bottom of each chip was scratched away to allow a contact to be made for the application of a back gate voltage. Each chip was glued into a 20-pin leadless ceramic package using conducting silver paint. Contact pads were bonded to the package pins with 25 µm diameter gold wires using an ultrasonic wedge bonder. The sample package was then inserted in to a spring-loaded holder at the end of a sample rod which fitted inside a commercial Oxford Instruments cryostat. A Cu-wound solenoid mounted on the tail of the cryostat was driven by a programmable bipolar Kepco power supply, generating a maximum magnetic field of 37.5 mT perpendicular to the plane of the Hall cross. A turbo pump was used to evacuate the sample space to ~10^−6^ mbar to remove adsorbed water molecules and other volatile surface contaminants and all measurements were made at ambient temperature.

Two-lead Resistance (*R*_*DS*_) - back gate voltage (*V*_*GS*_) measurements were performed using a Stanford Research Systems SR830 digital lock-in amplifier to provide a 10 µA, 32 Hz AC current and detect the resulting AC voltage, and a Source Measure Unit (SMU) provided the back gate voltage. A Keithley 2450 (SMU) in 4-wire configuration was employed to characterise the Hall coefficient and Hall offset of devices using a 10 µA DC current. The Hall voltage noise was characterised using a battery-driven DC current source and an ultra-low-noise preamplifier with 10^4^ gain, whose output was recorded at set Hall currents using a HP3561A dynamic signal analyser (DSA). Noise spectra were measured in the range 1 Hz – 1 kHz with a bandwidth of 1 Hz and averaged 100 times to reduce scatter.

## Data Availability

All data captured in the course of this research work are openly available from the University of Bath Research Data Archive at 10.15125/BATH-00587.
